# Restoration of the Dopamine Transporter through Cell Therapy Improves Dyskinesia in a Rat Model of Parkinson’s Disease

**DOI:** 10.1371/journal.pone.0153424

**Published:** 2016-04-14

**Authors:** D. Tomas, D. Stanic, H. K. Chua, K. White, W. C. Boon, M. Horne

**Affiliations:** 1 Florey Institute of Neuroscience and Mental Health, University of Melbourne, Parkville, Australia, 3010; 2 Department of Medicine, University of Melbourne, St Vincent’s Hospital, Fitzroy, Victoria, Australia, 3065; Karolinska Inst, SWEDEN

## Abstract

The dyskinesia of Parkinson's Disease is most likely due to excess levels of dopamine in the striatum. The mechanism may be due to aberrant synthesis but also, a deficiency or absence of the Dopamine Transporter. In this study we have examined the proposition that reinstating Dopamine Transporter expression in the striatum would reduce dyskinesia. We transplanted c17.2 cells that stably expressed the Dopamine Transporter into dyskinetic rats. There was a reduction in dyskinesia in rats that received grafts expressing the Dopamine Transporter. Strategies designed to increase Dopamine Transporter in the striatum may be useful in treating the dyskinesia associated with human Parkinson's Disease.

## Introduction

The principle motor sign of Parkinson’s Disease (PD) arises because neurodegeneration of Substantia Nigra pars compacta (SNpc) neurones and their axons, results in failing dopamine (DA) neurotransmission in the dorsal striatum. At the onset of disease, treatment with levodopa improves motor function, but within 5 years this beneficial effect is marred by “wearing off” in about 50% of patients. “Wearing off” [1–5] refers to the increasingly shorter duration of efficacy of a single administration of levodopa, which eventually comes to mirror the plasma levels of levodopa [2,6]. The explanation provided for “wearing off” is that the physiological capacity to store DA, which is rapidly synthesised by Aromatic Amino Acid Decarboxylase (AADC), is eroded so that newly synthesised DA is immediately delivered into the synaptic space and is available for neurotransmission. As synthesised DA is normally stored in vesicles of DA terminals, the progressive loss of DA terminals in PD is the most likely explanation for loss of storage. Dyskinesia almost invariably accompanies “wearing off”, and the threshold and time-course for dyskinesia comes to parallel the anti-Parkinsonian effect of levodopa [1,7–10].

In a previous study, we examined the events leading to the loss of dopamine storage and the emergence of dyskinesia [11] and briefly summarise these findings here. We found that in normal rats, there was either a modest, or no rise in striatal DA concentration ([DA], measured by dialyses) following administration of levodopa, presumably because of rapid clearance of DA from the synaptic space by re-uptake through the dopamine transporter (DAT). When lesions of varying size are made to the rat SNpc, DAT expressing DA terminals (and hence DAT) only begin to fall when the lesion size is greater than ~70% (see also [11–15]). Furthermore, [DA] in the striatum following administration of levodopa only began to increase when the number of DAT expressing terminals in the striatum began to fall and dyskinesia (as measured by Abnormal Involuntary Movements Scale: AIMS) increased linearly with the increase in striatal [DA]. AIMS increased sharply when less than 20% of DAT expressing terminals remain (S1 Fig). Lee et al [11] proposed that the reason that [DA] and dyskinesia increased sharply was due to loss of DAT function resulting from the loss of DAT expressing fibres. On the basis of this previous study[11], we conclude that reinstating even a small amount of DAT expression in the striatum might significantly ameliorate dyskinesia. DAT is reduced in dyskinetic rats, non-human primates [11,15], and in people whose PD is complicated by dyskinesia [16]. A previous study demonstrated that grafting dopaminergic neurons expressing DAT reduced dyskinesia more than grafts of dopaminergic neurons that did not express DAT[17]. In this study we tested the proposition that a modest increase in DAT expression would significantly reduce levodopa related dyskinesia in rats by grafting cells that express DAT into the striatum of dyskinetic rats. A similar technique was used to show that expressing DAT in C17.1 cell grafted into the rodent striatum reduced their alcohol consumption. We chose these cells in this study because the previous study demonstrated that these cells could maintain stable expression of DAT after transfection and because these cells were of mouse origin which has advantage for immunohistochemistry if the rat is the host. The findings reported here suggest that increasing DAT expression in the striatum as means to treating dyskinesia is worthy of further investigation.

## Methods

All methods conformed to the Australian National Health and Medical Research Council published code of practice for the use of animals in research and were approved by the ethics committee of the Florey Institute for Neurosciences and Mental Health. Thirty-five male Wistar rats weighing 250–275 g were used.

### Generation of DAT expressing construct

The full length rat DAT cDNA was amplified from rat stratium total RNA (extracted using Qiagen RNeasy kit, Chadstone, Australia) by high fidelity RT-PCR (Transcriptor HF, Roche, Castle Hill, Australia; Phusion HF DNA polymerase, Finnzymes, Espoo, Finland; GC buffer; primers). PCR primers were designed based on rat DAT NCBI Reference Sequence NM_012694.2; forward primer includes the EcoRI and Kozak sequences before the start codon 5’CGG^AATTCTGCCACC**ATG**AGTAAGAGC 3’ whereas the reverse primer includes the XbaI downstream of the stop codon 5’GCCTAGT^CTAGACACT**TTA**CAGCAACAGCC 3’.

The amplified rat DAT cDNA (1869bp) was cloned into EcoRI/XbaI sites of the pcDNA3.1/Zeo vector (Invitrogen, Carlsbad, CA) with expression under the control of CMV promoter. The identity of the cloned amplicon was confirmed by Sanger sequence analysis (Applied Biosystems 3130xl Genetic Analysers, MHTP Medical Genomics Facility, Australia).

### Cell lines

C17.2 cells (an immortalised mouse neural progenitor cell line derived from postnatal mouse cerebellum[18]) was transfected with either rat DAT construct (DAT cells) or vector control plasmid (Null cells) as described above[19]. Selection pressure was asserted by addition of the antibiotic Zeocin (Invitrogen) to the complete DMEM media. Briefly, 48h after transfection, C17.2 cells were passaged into 12-well plates, in 1:10, 1:20, and 1:40 dilutions, and cultured in selection medium (i.e. complete DMEM containing 400 μg/mL Zeocin) for 2 weeks. Media were changed every 2 days. Only cells expressing the pcDNA3.1/Zeo(+) plasmid would be resistant to Zeocin toxic effect. Each confluent well (i.e. each stable line) was expanded into T25 flasks with selection medium. At 80% confluency, two-thirds of the cells were harvested and frozen into aliquots and one-third of the cells were passaged into new T25 flask. The selection process was repeated 13 times; cells were harvested and frozen down after each selection cycle. Total RNA and protein were extracted from harvested cells using the Paris kit (Ambion®, Life Technologies, Mulgrave, Australia) and DAT expression was analysed by RT-PCR according to manufacturers’ instructions (RT: Superscript II Reverse Transcriptase/random primers, Life Technologies; PCR: GoTaq Green/rDAT primers, Promega) and Western Blot analyses. DAT cell lines that continuously expressed DAT transcript and protein in all selection rounds were expanded and frozen in liquid nitrogen. Null cell lines that survived 13 rounds of selection were also expanded and frozen in liquid nitrogen. For transplantation, frozen cell aliquots were expanded using the selection medium. Harvested DAT or Null cells were rinsed three time in Earle's Balanced Salt Solution (EBSS, Gibco, USA), and resuspended in EBSS to a density of 8.5X10^4^ cells/μl for grafting.

### Surgery and dyskinesia

**Fig 1:**
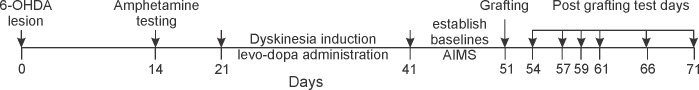
A time-line of the experiments. A time line showing the interval between each experimental step.

Anesthesia was induced by inhalation of 5% isoflurane (Delvet, Seven Hills, NSW, Australia), and maintained with 1.5% isoflurane through a nose cone. Large (> 90%) unilateral lesions of the nigrostriatal pathway were made in rats by injecting 4 μl of a 3.5 μg/μl solution of 6-hydroxydopamine (6-OHDA; Sigma-Aldrich, St. Louis, MO) in 0.9% NaCl containing 0.02% ascorbic acid, into the right medial forebrain bundle at 4.4 mm caudal and 1.2 mm lateral to Bregma, and 7.8 mm below dura (Paxinos and Watson, 1998). Fourteen days later (see timeline in Fig 1), the number of rotations in the 90 mins following administration of amphetamine (2.5-5mg/kg i.p) was counted as an initial estimate of the extent of nigral cell loss: lesions that resulted in more than 90% loss of dopaminergic cells were expected to produce more than 5 rotations per min.

Twenty-one days after 6-OHDA administration, levodopa (levodopa methyl ester hydrochloride (Sigma, #D1507) and benserazide (Sigma, #B7283) (6 and 15 mg/kg, respectively, i.p), in 0.9% saline and 0.02% ascorbic acid), was administered daily for 20 days to induce dyskinesia, and then animals were scored daily until scores were stable (which occurred by day 30 at the latest). Dyskinesia was scored was using the AIMS [11,20,21]. AIMS scoring followed levodopa administration as follows. Animals were placed individually in transparent plastic cages with bedding material and forelimb, orolingual, axial; and locomotive behaviour was scored over one minute as: 0, absent; 1, present less than 50% of the time; 2, present more than 50% of the time; 3, continuous but suppressible by a strong startling stimulus; and 4, continuous and uninterruptible by strong startling stimuli. Scoring was performed every 20 min, for 180 mins (i.e. 9 occasions for each administration of levodopa). Thus the maximum score at each measurement was 16 and for each administration was 144 (16 x 9). Once scores were stable, AIMS from 4 separate days were averaged to produce the pre-grafting level of dyskinesia.

Rats were then anesthetized with isoflurane (as above), holes drilled in the skull over injection sites, and a glass micropipette attached to a 10 μl Hamilton syringe was placed at the following coordinates: 1.0 mm rostral and 2.5 mm lateral to Bregma, and 4.5 mm and 4.0 mm below dura; and 0.2 mm anterior and 3.0 mm lateral to Bregma, and 4.5 mm and 4.0 mm below dura. Each of these four locations were injected with 1x10^5^ cells suspended in 1 ul of buffer (~84,000 cells), at a rate of 1 μl/min. The micropipette was left in place for 2 minutes following each injection before removing it from the brain. The cells were kept on ice until transplanted. Daily levodopa administration recommenced the day following surgery and continued until the end of the study (day 20), with AIMS scores score assessed according to the schedule in the Results section. All grafted animals received a daily injection of cyclosporin A (20mg/kg, LC laboratories, Woburn, MA, USA) commencing the day before transplantation and continuing until animals were killed at end of experiment.

### Tissue preparation

Animals were deeply anaesthetized using pentobarbitone sodium (Lethabarb, Virbac, Milperra, NSW, Australia; 100mg/kg i.p.) and transcardially perfused with Phosphate Buffered Saline (PBS) at 37°C, followed by 4% paraformaldehyde (Sigma) and 0.2% picric. The brains were dissected out and placed in the same fixative for 90 min at 4°C, and finally immersed for 48h at 4°C in 30% sucrose dissolved in 0.1 M phosphate buffer Saline (PBS). The brains were snap frozen and then cut in coronal sections using a cryostat (Leica CM1850 (Leica CM1850, Wetzlar, Germany) at a thickness of 14 μm, thaw-mounted on slides coated with 0.5% gelatin (Sigma) and 0.05% chromium(III) potassium sulphate dodecahydrate (Merck, KGaA, Darmstadt, Germany), and stored at -20°C.

### Immunohistochemistry

Antigen retrieval was performed where sections were rinsed in 0.1 M phosphate buffered saline (PBS), followed by 0.21% Citrate Buffer (pH 6.0) for 120 min at 90°C. Sections were then cooled to room temperature and rinsed (3 × 10 min) in 0.01 M PBS, followed by incubation in rat anti-DAT antibody (1:1000; Merck Millipore, Billerica, MA, USA; #MAB369) diluted in 0.01 M PBS, 0.3% Triton X-100 and 1.0% NRS for 24h at room temperature. Sections were then incubated in blocking diluent [0.01 M PBS containing 5% normal rabbit serum (NRS) and 0.3% Triton X-100 (Sigma)] for 30 min, biotinylated rabbit anti-rat (1:500; Vector, Burlingame, CA, USA) diluted in 0.01 M PBS and 1.0% NRS for 2h at room temperature, and then in avidin peroxidise (1:1000 in 0.01 M PBS; Sigma) for 1 h, followed by diaminobenzidine (1:100; DAB, Sigma) for 20 min. Hydrogen peroxidase (Merck) was added (0.01%) to the DAB solution for substrate precipitation and the reaction terminated 1–2 min later by rinsing sections in 0.01M PBS.

For fluorescence imaging, sections were also put through the above mentioned antigen retrieval process then double labeled with primary antibodies; Rat Anti-DAT antibody (1:500; Merck Millipore, USA; #MAB369) and Rabbit FOS-B (102) antibody (1:50; Santa Cruz; #SC-48) for 24 hours. Sections were then rinsed with 0.1M PBS and blocked with diluent [0.01 M PBS containing 5% normal goat serum and 0.3% Triton X-100] for 30 minutes. Secondary antibodies were applied for a further 2 hours at a dilution of 1:100, (goat anti-rabbit alexa fluor 488, and goat anti-rat alexa fluor 594 (Jackson Immuno Research). The sections were further rinsed with 0.1M PBS, then cover slipped using Dako fluorescent mounting medium. Identical exposure and gain settings were used to capture the images.

### Cell Quantification

The grafted region was identified by the presence of DAT-immunoreactive cell bodies/fibres, DAT-negative cell bodies, and/or a needle track associated with the grafting procedure. DAT-immunoreactive cell bodies were counted on 14 μm-thick sections, each 140 μm apart. Estimates of the number of grafted DAT-immunoreactive cells were made using a fractionator sampling design according to optical dissector rules [14,22–25]. The entire grafted area in each section was delineated, and regular predetermined x, y intervals (x = 150 μm; y = 110 μm) and counting frame dimensions (x = 150 μm; y = 110 μm) frame dimensions for all estimates were derived by means of a grid program (Stereoinvestigator v.7.0, MicroBrightField, Williston, VT, viewed through a microscope, Leica).

## Results

Thirty-five animals received unilateral lesions of the nigrostriatal pathway that produced more than 5 rotations per min following amphetamine but 6 of these had average AIMS scores prior to grafting that were less than 6, so these rats were also excluded from the study. The average AIMS score of the remaining 24 was greater than 50 in 16 animals (high dyskinesia group) and between 6–25 in the remaining 8 (low dyskinesia group)—see Table 1. These rats received either C17.2 cells that stably expressed DAT (DAT grafts, n = 15) or C17.2 cells transfected with the empty vector (Null grafts, n = 9) and the graft types were similarly distributed amongst the high and low dyskinetic animals (Table 1).

**Table 1:** Animals Used in the study.

			Graft Type	
	No. animals	Ave AIMS	Null	DAT
Number lesioned	35	-	-	-
Failed Lesion	13	1	-	-
Low AIMS (6–18)	6	12	2	4
High AIMS (45–78)	16	61	9	7

### Histological Assessment of the grafts

**Fig 2:**
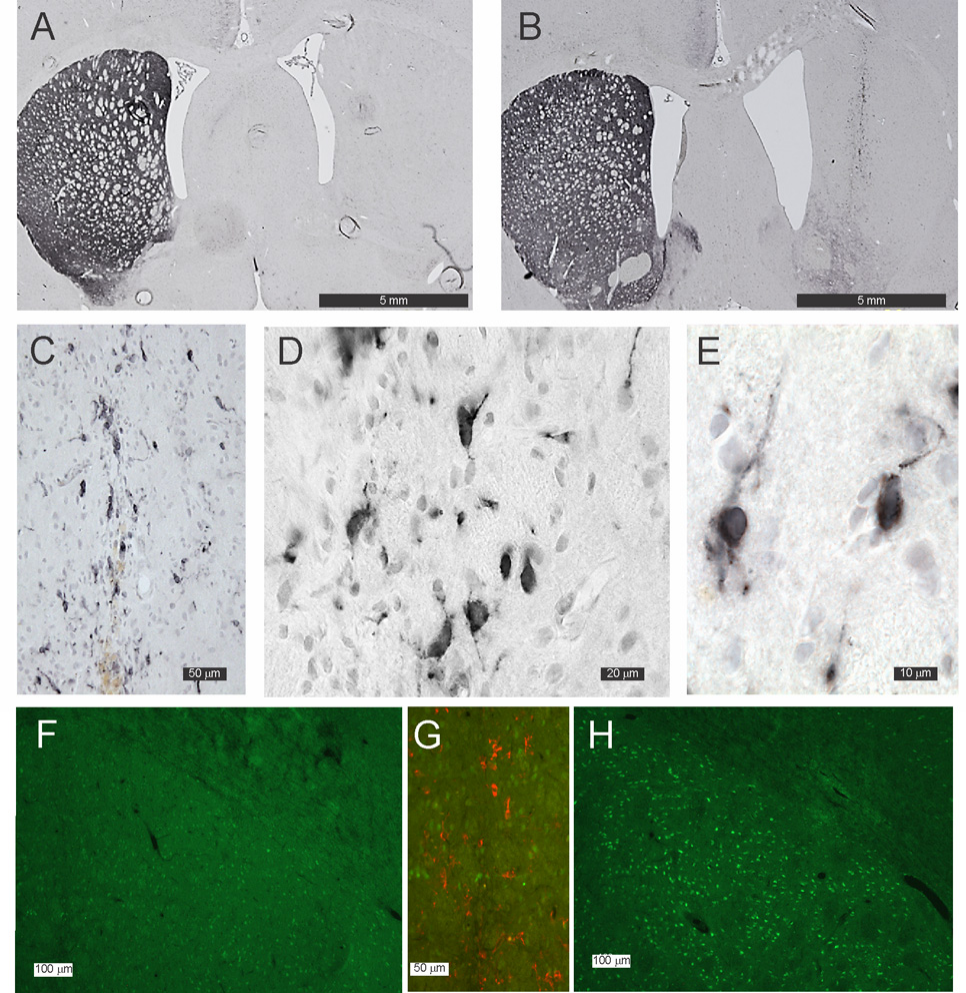
Photomicrographs of DAT-immunoreactivity in the striatum of grafted animals. (A) Null graft located in the striatum of a rat in whom 6-OHDA had been used to denervate the striatum. The graft tract is faintly discernible but there are no DAT-immunoreactive cell bodies, terminals or fibres. (B, C, D and E) show a DAT graft located in the striatum of a rat in whom 6-OHDA had been used to denervate the striatum; note the numerous DAT-immunoreactive cell bodies within the graft (B and D). (D and E) show DAT-immunoreactivity is present in the cytoplasm and along the numerous processes emanating from the cells. (F, G and H) show Fos- B immuno-reactivity (green) in a DAT grafted striatum (F) and a Null grafted striatum (H). (G) shows both Fos_B and DAT immuno-reactivity (red) merged in the one image in the region of the DAT graft shown by the white rectangle in (F).

Rats were killed 22 days after grafting was performed, followed by subsequent histological and stereological examination of the grafts (Table 2 and Fig 2). DAT-immunoreactive fibres and terminals in the striatum of animals that received Null grafts were either scanty or completely absent (Fig 2A). No DAT-immunoreactive cells were observed in the striatum of any of the Null grafted animals. The Null graft itself was often only discernable by the location of the injection tract. Qualitatively, low dyskinesia animals had more DAT-immunoreactive fibres/terminals in the denervated striatum than highly dyskinetic animals.

**Table 2:** Numbers of DAT-immunoreactive cells in grafted rats^*^.

	Null Grafts		DAT Grafts	
	High AIMS	Low AIMS	High AIMS	Low AIMS
Median	0	0	312	124
IQR	0	3.5	187	24
N	9	2	7	4

* No DAT-immunoreactive cells were seen in the striatum of Null grafted rats

**Fig 3:**
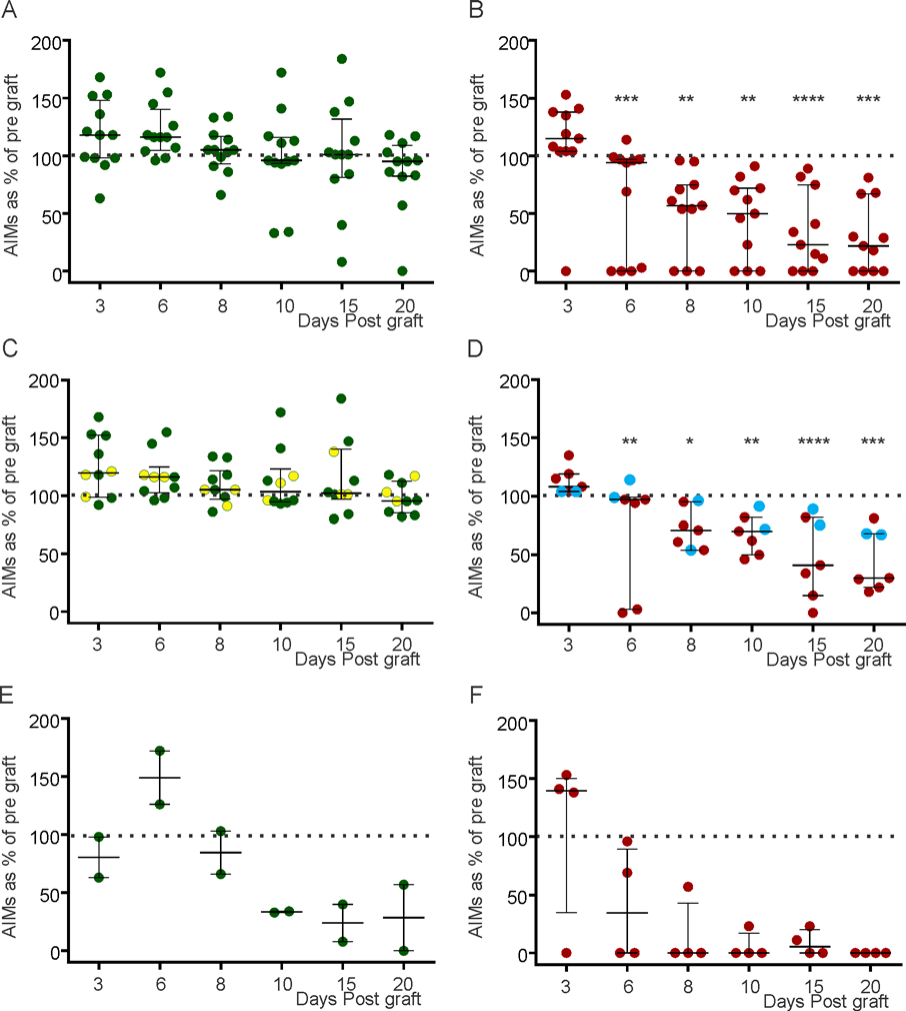
The percent change in AIMS (from pre-graft levels at various days post graft. In all figures, the y axis shows percent change in AIMS from pre-graft levels at various days post graft (x axis), bars show median and interquartile range and the statistical tests are ANOVA and Dunnet’s multiple comparisons tests where *, ** and *** indicate p<0.05, <0.01 and <0.001 respectively. (A) High and low dyskinetic animals that received Null grafts or “failed grafts. There was no significant difference between pre-graft AIMS and AIMS on any day post graft. (B) Highly and low dyskinetic animals that received a DAT graft. There was a significant difference between pre-graft AIMS at all days post-graft except day 3. (C) Animals with high AIMS (high dyskinesia group) that received a Null graft. The yellow circles represent the three “failed grafts” that were not included in calculations of significance. Note that one data point (243% on Day 3) is not shown. (D) Animals with high AIMS (high dyskinesia group) that received a DAT graft. The blue circles represent two animals with “eccentric grafts”. Note a steady and progressive fall of the median levels over the 20 days. (E) Animals with low AIMS (low dyskinesia group) that received a Null graft. Note the rapid recovery of AIMS plateauing at ~30% of pre-graft levels by day 10. (F) Animals with low AIMS (low dyskinesia group) that received a DAT graft. Note the rapid recovery of AIMS with median levels at 0% of pre-graft levels by day 8.

DAT-immunoreactive fibres/terminals in the striatum of animals that received DAT grafts were also either scanty or completely absent (Fig 2B). In the case of one DAT graft recipient, DAT-immunoreactive terminal were more obvious than all other lesioned animals. This rat was in the low dyskinesia group and referred to below as “the partially denervated DAT grafted animal” (see below). The DAT graft was readily discernable by the presence of DAT-immunoreactive cells along the injection track (Fig 2B and 2C). DAT-immunoreactivity was observed in the nucleus of these cells, but even more intensely in the cytoplasm (Fig 2D and 2E). There were numerous DAT-immunoreactive processes emanating from these cells. This pattern of immunoreactivity suggested that some of the DAT was located on cell membranes. Most of the DAT grafts were located in the dorsal striatum. However, the number of DAT cell in two grafts were low and in one animal the number of DAT-immunoreactive was quite high but they were located mostly in the overlying corpus callosum and cortex and with no cells in the striatum. These three cases have been analyzed separately and will be referred to as cases of “failed grafts” (Table 2 and Fig 3C). A further two grafts were located in the striatum, but one was more ventral and posterior with part of the graft in the globus pallidus and the other was in the most rostral part of the striatum (referred to below as “eccentric DAT grafted animals”).

As previously reported[26], there was a marked upregulation of Fos B immunoreactivity in neurons in the dorsolateral striatum of lesioned animals treated with L-DOPA, which was apparent in the Null grafted straita (Fig 2H), but not in the DAT grafted striata (Fig 2F and 2G).

### Change in AIMS scores following grafting

Prior to grafting, the average AIMS scores in the high dyskinesia group was 61±9.5 (mean ± SD) and 15±4 in the low dyskinesia group. In the high dyskinesia group that received DAT grafts, the averaged AIMS scores was 62±9 and in those that received Null grafts was 66±15. Thus prior to grafting, the AIMS scores were similar, implying similar degree of dopaminergic terminal denervation.

Daily levodopa administration recommenced the day following grafting, with AIMS scores assessed on days 3, 6, 8, 10, 15 and 20 post-graft. For each animal, the scores on each day were expressed as a percentage change from the average pre-grafting AIMS scores for that animal (Fig 3). The AIMS scores of animals receiving Null grafts increased transiently in most animals but was sustained over the whole 20 days in a few animals (Fig 3A). Over the 20 post graft days, the median AIMS scores had returned to 100% by day 10 and had fallen to 90% of pre-surgery level by day 20: this trend was not significant.

In animals receiving DAT grafts, there was a transient increase in AIMS scores on day 3 but the median AIMS scores fell rapidly to be 30% of pre grafting by day 20 (Fig 3B). Examination of Fig 3B shows that the AIMS scores fell rapidly in some animals and more slowly in others but overall was statistically different from pre graft AIMS scores at all days post graft except day 3 (ANOVA and Dunnet’s multiple comparisons test).

The data was further analyzed to establish whether cases with more rapid recover could be explained by the level of lesioning, using the post-lesioning AIMS scores and the extent of remaining DAT-immunoreactive fibres/terminals as a surrogate marker of the extent of striatal innervation. Thus animals that had high level of dyskinesia were examined separately to those with low levels of dyskinesia Fig 3C and 3D). Post-grafting AIMS scores in animals with high dyskinesia receiving Null grafts fell only minimally over the 20 days post-grafting (Fig 3C). AIMS scores of animals with failed DAT grafts were similar to the null grafted animals (yellow circles, Fig 2A). However, the AIMS scores of Null grafted animals with low dyskinesia fell over the 20 days post-grafting (Fig 3E), and these animals tended to have higher numbers of DAT-immunoreactive fibres/terminals in their striata.

In the case of the DAT grafted animals with high dyskinesia, AIMS scores were at pre-graft levels by day 6 and continued to fall over the 20 days post-grafting (Fig 3E). The trend was significant (p<0.0001, one way ANOVA) and was significantly less at all post-grafting days other than day 3 (Dunnet’s multiple comparisons test, Fig 3E). The two “eccentric DAT grafted animals” are included in this analysis but are shown as blue circles in Fig 3E. In these two animals AIMS scores did not fall as much as the other DAT grafted animals over the course of the 20 days. Indeed, a linear regression curve fitted to the means of each post-graft day (r^2^ = 0.95) indicates that the AIMS scores fell by 5% for each post graft day. The AIMS scores of DAT grafted animals with low dyskinesia also fell over the 20 days post-grafting, and were close to absent by 8 days in most animals (Fig 3F). These animals had more DAT-immunoreactive fibres/terminals in their striata, in particular “the partially denervated, DAT grafted animal”. There was no correlation between the extent of improvement in AIMS at 20 days post grafting and the number of DAT expressing cells found in the striatum.

## Discussion

This study examined whether reintroducing DAT expression, by grafting cells that express DAT into the striatum of dyskinetic rats, would reduce dyskinesia. In a previous study [11], we showed that there was a marked rise in striatal [DA] following administration of levodopa in dyskinetic rats. In that study, we provided evidence that because of a loss of DAT in dyskinetic animals, the clearance of extra synaptic DA is mostly likely by bulk diffusion. Thus, for this study we proposed that replacement of DAT would be effective in reducing dyskinesia as measured by AIMS scores in 6-OHDA lesioned rats treated with Levodopa. The main finding of this study is that AIMS scores fell significantly and progressively over the course of 20 days in animals receiving DAT grafts but in those animals receiving Null grafts there was no change in AIMS scores.

This finding was most apparent in animals with high levels of dyskinesia at the time of grafting; a comparison of Fig 3C and 3D, shows very clearly that there is a steady decline in AIMS scores over 20 days, in DAT grafted animals. Furthermore, the finding that ectopically placed grafts had no effect on AIMS scores (Fig 3C) argues that DAT expressing cells in the striatum are required to reduce dyskinesia. The finding that eccentrically placed grafts, which resulted in fewer DAT-immunoreactive cells at the centre of dorsal striatum were less effective in reducing AIMS scores, suggests that the number of DAT expressing located in the dorsal striatum (Fig 3E) will dictate the extent of AIMS scores reduction. S2 Fig lends support to the notion that the “dose” of DAT provided by the graft will determine the rate and possibly the extent of recovery. This may be relevant if this model were to be extended to therapeutic concepts because excess removal of dopamine by a high dose of DAT may result in bradykinesia as well as ameliorating dyskinesia. This potential risk of overexpression of DAT would be best tested in non human primates where the bradykinesia can be more readily assessed. There is also a risk in using cell that do not normal transport dopamine into the cell that the dopamine will cause toxicity [27]. While the possibility that a proportion of grafted DAT expressing cells degenerated because of dopamine toxicity cannot be discounted, it was not significant enough to obscure the observed improvement in dyskinesia. Future studies over longer periods would need to be mindful of this problem however. Cells such as C17.1 may have a trophic effect on cells or fibres in the grafted region [28]. However this does not appear to have been a significant influenced dyskinesia in this study because there was no improvement in dyskinesia in animals receiving Null grafts (ie grafts of C17.1 cells without DAT expression).

Rats from the low dyskinesia group receiving Null grafts appeared to recover spontaneously. Spontaneous recovery of animals can occur by sprouting and even with large lesions there is recovery over 16 weeks [15]. It is thus possible that some of the spontaneous improvement in dyskinesia seen in the “low dyskinesia” animals was due to sprouting of remnant fibres. It is relevant to this proposal occasional DAT positive fibres were more prevalent in these same animals than in the high dyskinesia group. All grafted animals described in this study had rotated at greater than 6/min in response to amphetamine. This suggest that the striatum had been extensively denervated. While there was suggestion that DAT-immunoreactive fibres were more frequent in the low rotators, this was clear-cut in only one animal (“the partially denervated DAT grafted animal”). There was a similar but more rapid recovery in the low dyskinesia group receiving DAT grafts. While sprouting is the most likely driver of recovery, the DAT grafts may have contributed to the more rapid recovery.

The initial worsening of AIMS scores following grafting suggests that levodopa treatment caused even greater striatal DA concentrations than prior to the grafts. The trauma of grafting may have resulted in a transient permeability of the vasculature and entry of catecholamines from the blood stream. It is important to note that the proposition that increased DAT expression improves dyskinesia, does not reflect on the putative sources of DA in the relative absence of DA terminals. Whether the DA is from serotonergic (5-HT) axons that express more AADC and thus have increased capacity to convert levodopa to DA [21] or other sources is immaterial to the question of how the peak levels associated with levodopa is attenuated.

In this study we use the number of DAT expressing cells as an indication of DAT expression. It might therefore be expected that the extent of improvement in AIMS might correlate with the number of DAT expressing cells found in the striatum. Dyskinesia was only modestly reduced in animals whose grafts were ectopic grafts and this observation is in keeping with their being a relation between relevant DAT expression and extent of dyskinesia. The reduction in dyskinesia was fairly similar in other animals and so a correlation would be difficult to discern. As discussed in the introduction, even a small increase in DAT expression has the potential to reduce dyskinesia. It is difficult to know the extent of DAT expressed on cell membrane surface by the C17.1 cells. However expression on the cell membrane may present a larger surface area expressing DAT than on fibres and terminals and furthermore the higher power figures show that many cells do have DAT expressing processes extending into the striatum. Whatever the case, the level of expression of DAT achieved by these cells has been sufficient to markedly reduce AIMS. As the only difference in treatment between the control group and the experimental arm was the expression of DAT, it is reasonable to propose that it was the expression of DAT that was the main agent reducing dyskinesia. A previous study also showed that the presence of DAT on grafts resulted in less levodopa induced dyskinesia[17]. In that study the presence of DAT did not have an adverse effect on the extent to which bradykinesia was reduced by levodopa. The potential risk that overexpression of DAT will cause bradykinesia would be best tested in non human primates where the bradykinesia can be more readily assessed.

Re-uptake of DA is impaired in dyskinetic animals (rats and non-human primates) [11,15] and humans [16]. PD is associated with loss of dopaminergic terminals in the striatum and it is likely that DAT is initially down-regulated in surviving terminals as a compensatory mechanisms. DAT will also be depleted simply by the attrition of terminals. The findings reported here suggest that only a modest increase in DAT expression in the striatum can reduce dyskinesia and is worthy of further investigation as a possible therapy. It is relevant that a similar approach was successful in reducing alcohol consumption in mice [19]. In summary, these findings suggest that smoothing levels of striatal DA by restoring DAT expression may be a useful therapeutic approach for treating dyskinesia.

## Supporting Information

S1 Fig Relationship between the extent of dyskinesia (AIMS) and the number of DAT positive fibres in the dorsal striatum. Data presented in Fig 2 of Lee et al [11] has been reanalyzed to show the relationship between the extent of dyskinesia (AIMS) and the number of DAT positive fibres in the dorsal striatum remaining in the striatum of the rat after administration of 6 OHDA (expressed as a percentage of the number in the unlesioned rat). The reader is referred to the original paper of Lee et al [11] for the full details of the method for this work.
(TIF)

S2 Fig Correlation between time and reduction in dyskinesia in grafted animals. This shows the same data as in Fig 2E, plotted against a linear scale and with two linear regression lines. The red line is a plot of regression line for the median of all data at each time point and shows that the AIMS score changes by 5% from pre grafting levels for every day post graft with an r^2^ = 0.95. The green line is a plot of regression line for the median of the eccentric cells at each time point and shows that the AIMS score changes by 2% from pre-grafting levels for every day post-graft with an r^2^ = 0.74.
(TIF)
