# Supplementary figures and images for: Restoration of the Dopamine Transporter through Cell Therapy Improves Dyskinesia in a Rat Model of Parkinson’s Disease
Source: PLoS One. 2016 Apr 14;11(4):e0153424. doi: 10.1371/journal.pone.0153424 (PMC4831749; doi:10.1371/journal.pone.0153424)

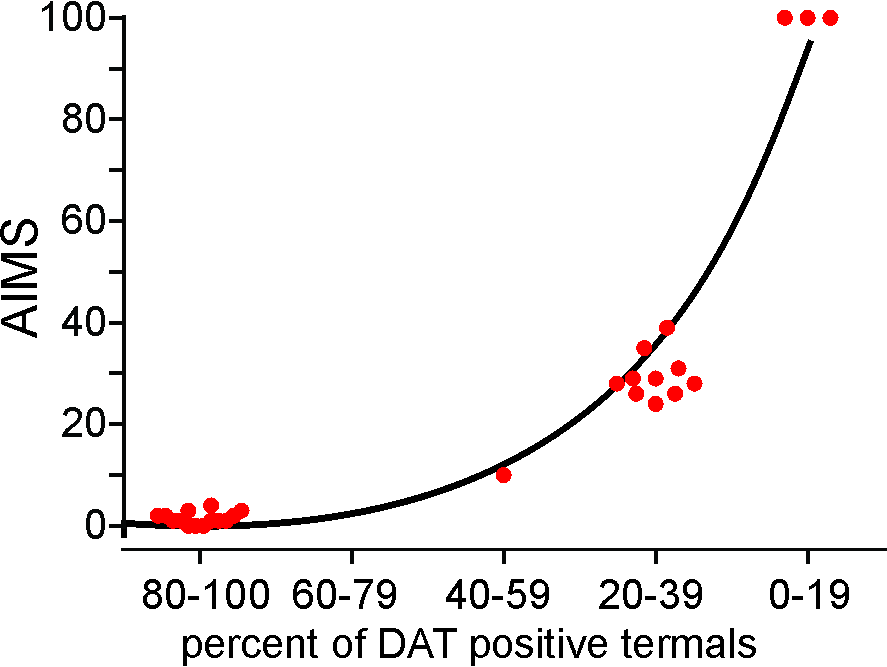

Supplement: S1 Fig — Data presented in Fig 2 of Lee et al [11] has been reanalyzed to show the relationship between the extent of dyskinesia (AIMS) and the number of DAT positive fibres in the dorsal striatum remaining in the striatum of the rat after administration of 6 OHDA (expressed as a percentage of the number in the unlesioned rat). The reader is referred to the original paper of Lee et al [11] for the full details of the method for this work. (TIF) [file pone.0153424.s001.tif]

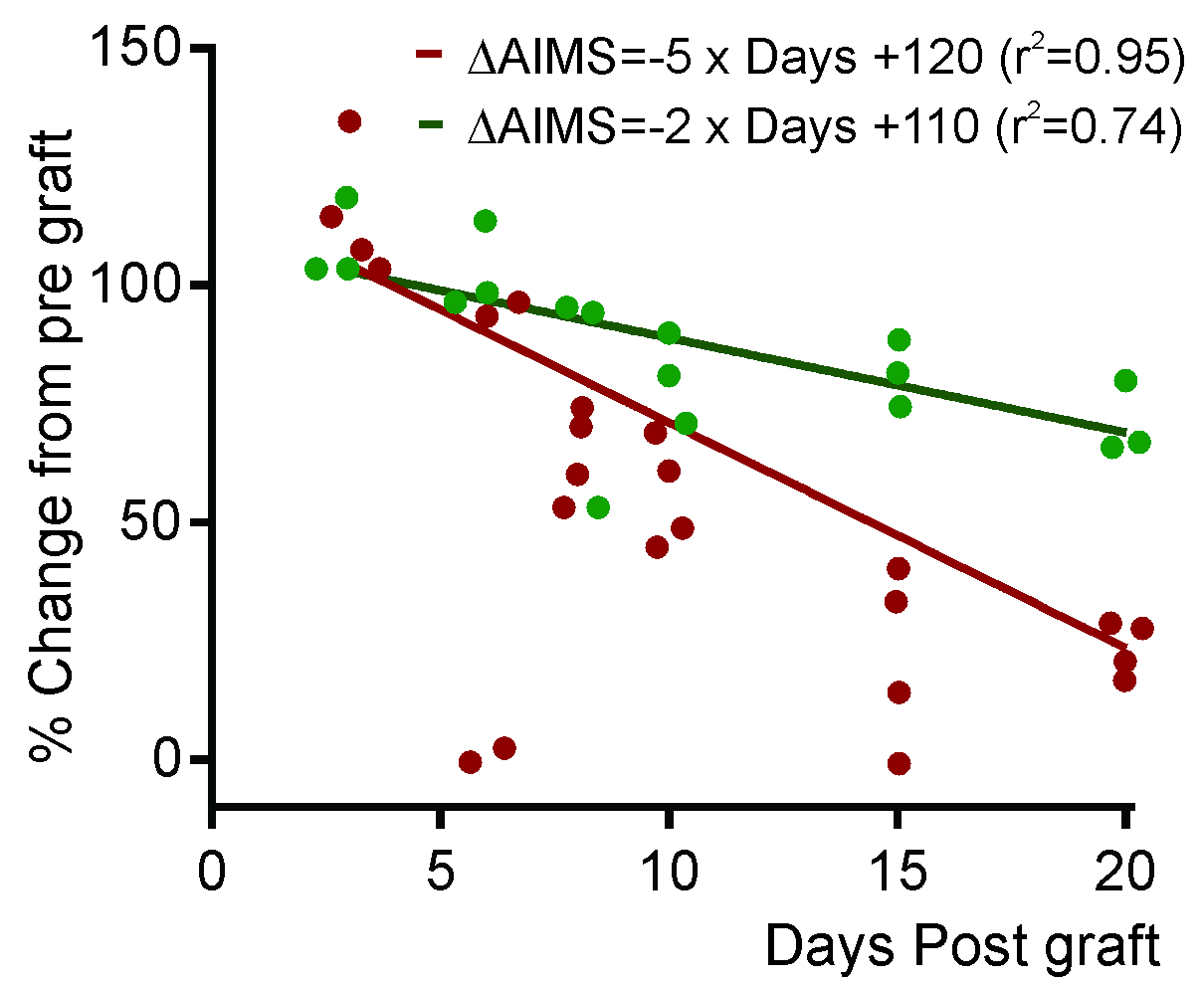

Supplement: S2 Fig — This shows the same data as in Fig 2E, plotted against a linear scale and with two linear regression lines. The red line is a plot of regression line for the median of all data at each time point and shows that the AIMS score changes by 5% from pre grafting levels for every day post graft with an r2 = 0.95. The green line is a plot of regression line for the median of the eccentric cells at each time point and shows that the AIMS score changes by 2% from pre-grafting levels for every day post-graft with an r2 = 0.74. (TIF) [file pone.0153424.s002.tif]
